# Unveiling the molecular landscape of δ-thalassemia and δ-globin variants in southern China: novel mutations, gene spectrum, and implications for thalassemia diagnosis

**DOI:** 10.3389/fgene.2025.1584310

**Published:** 2025-05-09

**Authors:** Youqiong Li, Lihua Ye, Liang Liang, Lihong Zheng, Yongjun Xiao, Zhongchan Lao, Jinping Bai, Xi He, Qixun Fang, Ting Qin

**Affiliations:** ^1^ Center for Medical Genetics and Prenatal Diagnosis, People’s Hospital of Guangxi Zhuang Autonomous Region, Nanning, China; ^2^ Department of Clinical Laboratory, Women and Children Care Hospital of Laibin, Laibin, China; ^3^ Department of Clinical Laboratory, The Second Nanning People’s Hospital, Nanning, China; ^4^ Department of Clinical Laboratory, Women and Children Care Hospital of Lingshan, Qinzhou, China; ^5^ Department of Research and Development, Yaneng Bioscience (Shenzhen) Co. Ltd., Shenzhen, China

**Keywords:** δ-thalassemia, δ-globin variants, *HBD* gene, Hb A_2_, thalassemia

## Abstract

**Objectives:**

δ-thalassemia and δ-globin variants are rare hemoglobinopathies. However, co-inheritance of β-thalassemia and δ-globin gene mutations may affect the diagnosis of β-thalassemia carriers when based on the elevated Hb A_2_. This study aimed to identify and characterize δ-thalassemia and δ-globin variants in Southern China.

**Methods:**

Ninety samples with suspected δ-globin gene mutations from 15,642 participants were selected for further molecular analysis based on their Hb A_2_ level (≦1.8%) and hematological parameters. Additionally, 37 samples with suspected δ-globin gene mutations were sent from other hospital to our laboratory for identification. GAP-PCR and PCR-reverse dot blot (PCR-RDB) were used to detect common α- and β-thalassemia in the Chinese population, and Sanger sequencing was used to identify δ-globin gene mutations.

**Results:**

Among 15,642 samples examined, samples with δ-globin gene mutations were identified in 127 (0.81%) cases with as many as 28 different genotypes, including 81 (0.52%) cases of δ-thalassemia and 46 (0.29%) cases of δ-globin variants. The most prevalent δ-thalassemia and δ-globin variants of this study were *HBD*:c.−127T>C (75.3%, 61/81) and Hb A_2_-Melbourne (54.3%, 25/46). Most of the samples were heterozygous (87.4%, 111/127), and only two cases of homozygous were detected. There were three double heterozygotes and 11 cases of combined α/β-globin mutations. Notably, we also identified eight cases of novel mutations in the δ-globin gene. In both heterozygous and homozygous cases, δ-globin mutations maintained hematological parameters within normal ranges, while their co-occurrence with α- or β-thalassemia manifested as a thalassemia phenotype characterized by significantly reduced MCV and MCH values.

**Conclusion:**

The study reveals that δ-globin gene mutations are prevalence in the South China and necessitates integration of δ-globin screening into existing thalassemia prevention protocols.

## Introduction

Hemoglobinopathies are the most common hereditary diseases in China, with a higher prevalence in region south of the Yangtze River (Huang et al., 2019; Chen et al., 2022; Wang et al., 2022). These disorders can be classified into two types: thalassemia and structural hemoglobin (Hb) variant. Thalassemia is characterized by a reduction or absence in the production of normal globin chains, while structural Hb variants are caused by amino acid substitutions in the globin chains (Viprakasit and Ekwattanakit, 2018; Vijian et al., 2021). α- and β-globin variants, along with thalassemia, are the most common and severe types of hemoglobinopathies (Lou et al., 2023; Paiboonsukwong et al., 2022). In contrast, δ-globin variant and thalassemia are less frequently reported, as the carriers of these conditions are less common in the population (Kordafshari et al., 2016; Morgado et al., 2007). The clinical presentation of these hemoglobinopathies can range from being asymptomatic to causing severe transfusion-dependent anemia accompanied by other complications (Harteveld et al., 2022). Therefore, early detection and accurate diagnosis are essential for preventing the development of severe forms of hemoglobinopathies.

Hb A_2_ level is a crucial hematological marker for distinguishing between α- and β-thalassemia carriers (Srivorakun et al., 2020; Colaco and Nadkarni, 2021). In normal individuals, Hb A_2_ accounts for less than 3.5% of total Hb and is composed of α- and δ-globin chains (α2δ2). Elevated Hb A_2_ levels (Hb A_2_>3.5%) are usually considered β-thalassemia carrier in Chinese clinical laboratories. Genetic defects in the δ-globin gene (*HBD* gene) can lead to a reduced Hb A_2_ level (Hanart et al., 2023). Our laboratory experience revealed that HbA2 values for δ-globin mutant heterozygotes ranged from 1.1% to 1.8% using the CE assay. Clinical implications are not associated with either structural Hb A_2_ variants (δ-globin variant) or δ-thalassemia caused by mutations in the δ-globin gene. However, co-inheritance of a mutation in the δ-globin gene and β-thalassemia might cause the phenotype of elevated Hb A_2_ characteristic of β-thalassemia carriers to decrease to normal or borderline level, thus causing diagnostic results to be misinterpreted (Panyasai and Pornprasert, 2020; Chen et al., 2017). In previous studies, we reported that δ-globin variants combined with β-thalassemia affected the diagnosis of β-thalassemia (Lin et al., 2024; Li et al., 2020). To improve the identification of β-thalassemia phenotype and at-risk couples in regions with high thalassemia prevalence, it is important to establish a regional database of δ-globin gene mutations.

In this study, we aim to identify the δ-thalassemia and δ-globin variants in southern China, based on reduced HbA_2_ levels as quantified by capillary electrophoresis. We also report eight novel mutations for the first time.

## Materials and methods

### Samples

The population of this study included 15,642 individuals who underwent routine screening for thalassemia in our hospital from January 2020 to December 2024. Out of a total of 15,642 samples processed, 90 samples were selected for further molecular analysis based on their Hb A_2_ level and hematological parameters. Based on our laboratory experience, an Hb A_2_ level of ≤1.8 can be used to as a screening criterion for δ-globin gene mutations after exclusion of other diseases (e.g., iron deficiency anemia). In addition, 37 samples with suspected δ-globin gene mutations were sent from outside hospitals to our laboratory for identification. This study was approved by the Ethics Committee of People’s hospital of Guangxi Zhuang Autonomous Region. Informed consents were collected from the participants.

### Hematological parameters and Hb analysis

The automated blood cell counters (Sysmex, kobe, Japan) were used to assess the hematological parameters of red blood cell counts. Hb fractions separation and quantification were carried out using by capillary electrophoresis (CE) system (Sebia capillarys2 Flex Piercing; Sebia, Paris, France). The reference range for normal hematologic parameters are mean corpuscular volume (MCV) 82∼100 fL and mean corpuscular Hb (MCH) 27∼35 pg. After excluding iron deficiency anemia, subjects with low Hb A_2_ levels (<2.4%) were considered α-thalassemia carriers, and ≤1.8% are suspected to be carriers of the δ-globin gene mutations. The reference interval is 2.4% < Hb A_2_ < 3.5%.

### Routine genetic test for thalassemia

Genomic DNA was extracted from peripheral blood according to the kit protocol (Yaneng Biotechnology Company, Shenzhen, China). The gap-polymerase chain reaction (Gap-PCR) was used to identify the four prevalent forms of deletional α-thalassemia in the Chinese population: --^SEA^, --^THAI^, -α^3.7^, and -α^4.2^ (Yaneng Biotechnology Company, Shenzhen, China). PCR and reverse dot blot (PCR-RDB) were used for determining the three common mutations of the α-globin gene: Hb Westmead (Hb WS), Hb Quong Sze (Hb QS), and Hb Constant Spring (Hb CS) (Yaneng Biotechnology Company, Shenzhen, China). The 17 known β-thalassemia mutations including −32 (C→A), −30 (T→C),−29 (A→G), −28 (A→G), CD14/15 (+G), CD17 (A→T), CD26 (G→A) (Hb E), CD27/28 (+C), CD31 (−C), CD41/42 (-TTCT), CD43 (G→T), CD71/72 (+A), IVS-Ⅰ-1 (G→T), IVS-Ⅰ-5 (G→C), IVS-Ⅱ-654 (C→T), 5′UTR+40–43 (−AAAC) (CAP), and Initiation codon (ATG>ACG) were analyzed by PCR-RDB (Yaneng Biotechnology Company, Shenzhen, China).

### Sanger sequencing of the δ-globin gene

Sanger sequencing of the δ-globin gene was performed to ascertain the existence of mutations in the gene. The amplification primers, conditions and system were as described in our previous reports (Li et al., 2020). The PCR fragments were sequenced by an 3500XL automated genetic analyzer (ABI, Foster City, CA, United States).

### Bioinformatic analysis

To assess the pathogenicity of novel δ-globin gene mutations, we employed three established computational prediction algorithms: PolyPhen-2 (probabilistic classification of missense variants), SIFT (Sorting Intolerant From Tolerant), and MutationTaster. PolyPhen-2, applicable exclusively to missense variants, generates normalized scores (0–1) reflecting deleterious potential, with classifications defined per db SNP/HGMD (The Human Gene Mutation Database) standards as benign (score ≤0.446), possibly damaging (0.447–0.908), or probably damaging (≥0.909). Parallel analysis using SIFT quantified amino acid substitution impacts through evolutionary conservation metrics, designating variants as deleterious (score ≤0.05) or tolerated (>0.05). MutationTaster provided complementary functional predictions through a Bayesian framework, categorizing variants into four clinically relevant classes: disease-causing automatic (A), disease-causing (D), polymorphism (N), or polymorphism automatic (P). This multi-algorithm approach aligns with ACMG/AMP guidelines for clinical variant interpretation and demonstrates critical utility in resolving ambiguous δ-globin variants lacking population frequency data.

## Results

### Prevalence of δ-globin gene

In this study, a total of 127 cases of δ-globin gene mutations were detected, including 37 samples sent to our laboratory from outside hospitals for characterization. In our hospital, 90 samples with δ-globin gene mutations were detected. Based on the number of screened cases, we can deduce that the carrier rate of the population in southern China is 0.81% (127/15,642), of which 0.52% (81/15,642) is in the case of δ-thalassemia, and the δ-globin gene variants is 0.29% (46/15,642). Of the 37 cases of δ-globin gene mutations sent from outside hospitals to our laboratory for detection, 17 were δ-thalassemia and 20 were δ-globin gene variants.

### Genotype and phenotype features of δ-thalassemia

**FIGURE 1:**
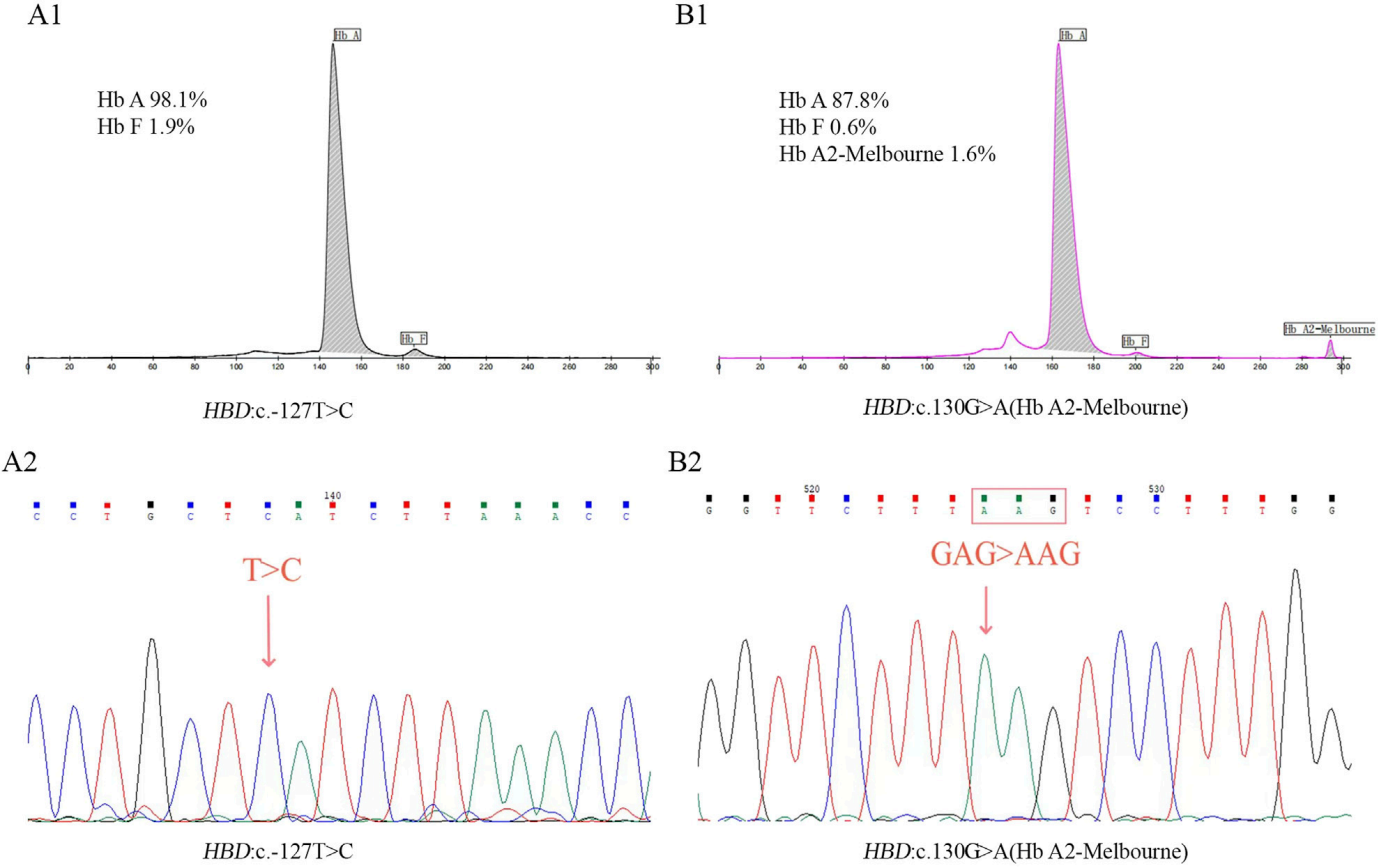
Results of CE **(A1)** and Sanger sequencing **(A2)** in homozygous mutation of *HBD*:c.-127T>C. Results of CE **(B1)** and Sanger sequencing **(B2)** in homozygous mutation of Hb A2-Melbourne.

This study identified 11 distinct mutations across 81 δ-thalassemia individuals, with an overall carrier rate of 0.52% (81/15,642) in the screened poplulation (Table 1). The most prevalent δ-thalassemia mutation was *HBD*:c.−127T>C, representing 75.3% (61/81) of cases, followed by *HBD*:c.-80T>C (n = 5, 6.2%). In the heterozygous mutation state, the hematological parameters were observed as follows: Hb 144.0 ± 16.9 g/L, MCV 89.5 ± 3.8 fL, and MCH 30.1 ± 1.8 pg. A significant reduction in Hb A_2_ levels (range: 1.1%–1.8%; 1.3% ± 0.1%) was characteristic of δ-thalassemia in heterozygous carriers. Only one homozygous case of δ-thalassemia (*HBD*: c.−127T>C) was identified in this study, with CE analysis demonstrating the absence of Hb A_2_ peak (Figure 1A).

**TABLE 1:** Hematological characteristics and Hb analysis of δ-thalassemia in this study.

Common name	HGVS name	Number	Hb (g/L)	MCV (fL)	MCH (pg)	Hb A (%)	Hb A2 (%)	Hb F (%)	IthaGenes ID
−77T>C	*HBD*:c.−127T>C	61	143.3 ± 18.0	88.3 ± 6.2	29.7 ± 2.7	96.9 ± 5.7	1.3 ± 0.3	1.0 ± 1.1	1322
−30T>C	*HBD*:c.−80T>C	5	137.4 ± 14.3	89.4 ± 3.1	30.1 ± 1.5	98.2 ± 1.0	1.4 ± 0.1	0.4 ± 0.9	1329
IVS I-127(−A)/IVS I-3(−A)	*HBD*:c.93-2delA	3	136.7 ± 24.0	86.7 ± 2.8	27.8 ± 1.7	98.6 ± 0.2	1.1 ± 0.1	0.2 ± 0.3	3233
CD 10 GCT>−CT	*HBD*:c.31delG	2	123.5 ± 6.4	86.8 ± 10.8	28.5 ± 6.4	98.5 ± 0.6	1.3 ± 0.3	0.3 ± 0.4	3232
CD7(−GAG)	*HBD*:c.22_24delGAG	1	132	91.3	29.5	98.8	1.2	0	3231
CD 97 CAC>CAT	*HBD*:c.294C>T	1	136	60	18.3	92	2.8	5.2	3787
CD 87 CAG>TAG	*HBD*:c.262C>T	2	145.5 ± 5.0	88.7 ± 0.3	30.2 ± 0	98.7 ± 0.1	1.3 ± 0.1	0	3844
CD122/123(+A)	*HBD*:c.369dupA	1	153	87.9	30.9	98.5	1.5	0	Unregistered
−84C>T	*HBD*:c.−134C>T	2	152 ± 14.1	87.4 ± 1.1	29.9 ± 0.4	98.2 ± 0	1.8 ± 0	0	Unregistered
Poly A+70 G>A	*HBD*:c.*200G>A	1	162	91.5	31.4	98	1.3	0	Unregistered
Int ATG>ACG	*HBD*:c.2T>C	2	145 ± 9.9	88.4 ± 2.4	29 ± 0.8	98.7 ± 0.1	1.35 ± 0.1	0	Unregistered

Reference ranges for parameters: Hb (115–155 g/L for females, 120–160 g/L for males), MCV (80–100 fL), MCH (27–34 pg), Hb A (91.5%–97.6%), Hb A2 (2.4%–3.5%), Hb F (0%–5.0%).

### Genotype and phenotype features of δ-globin variants

Seventeen different mutations were found in 46 δ-globin variants in this investigation, and the population that was screened had an overall carrier rate of 0.29% (46/15,642) (Table 2). Among δ-globin variants, Hb A_2_-Melbourne (*HBD*: c.130G>A) was the most frequent (n = 25, 54.3%), while other variants such as Hb A_2_-Coburg and Hb A_2_-Henan occurred in smaller cohorts (n = 2–3). In CE analyses, the majority of δ-globin variants are characterized by resolvable Hb A_2_ variants, while six variants remained undetectable. A homozygous case of Hb A_2_-Melbourne was identified, with CE revealed the lack of a visible Hb A_2_ peak (Figure 1B). Normal hematological parameters were preserved by δ-globin variants in both heterozygous and homozygous situations.

**TABLE 2:** Hematological characteristics and Hb analysis of δ-globin variants in this study.

Hb name	HGVS name	Number	Hb (g/L)	MCV (fL)	MCH (pg)	Hb A (%)	Hb A_2_ (%)	Hb F (%)	Variant (%)	IthaGenes ID
Hb A_2_-Melbourne	*HBD*:c.130G>A	25	136.2 ± 22.5	85.7 ± 9.8	28.0 ± 4.3	95.8 ± 8.9	1.4 ± 0.4	0.2 ± 0.4	1.0 ± 0.4	1364
Hb A_2_-Coburg	*HBD*:c.350G>A	2	153.5 ± 2.1	95.6 ± 10.1	29.2 ± 0.1	98.0 ± 0.5	1.4 ± 0.1	0.2 ± 0.3	0.7 ± 0.4	1385
Hb A_2_-Henan	*HBD*:c.221A>T	2	146/N	89.1/N	28/N	96.8 ± 0.4	1.7 ± 0.1	0.3 ± 0.4	1.3 ± 0.1	3335
Hb A_2_-Huadu	*HBD*:c.127T>C	2	122.5 ± 12.0	88.7 ± 3.9	28 ± 1.1	98.6 ± 0	1.4 ± 0	0	0	3239
Hb A_2_-Lepore	*HBD*:c.350G>T	2	162 ± 1.4	93.2 ± 4.0	30.9 ± 1.8	97.7 ± 0.4	1.4 ± 0.1	0	1.0 ± 0.4	3318
Hb A_2_-Guangxi*	*HBD*:c.238G>A	2	162	91.5	31.4	98	1.3	0	0.7	4091
Hb A_2_-Troodos	*HBD*:c.349C>T	1	134	86.1	29.7	97.7	1.4	0	0.9	1384
Hb A_2_-Fengshun	*HBD*:c.364G>A	1	N	N	N	97.3	1.4	0.5	0.9	3058
Hb A_2_-Yulin	*HBD*:c.139G>A	1	109	57.1	18.9	91.9	2.7	4	1.4	4069
Hb A_2_-Liangqing	*HBD*:c.362A>C	1	160	88.6	28.9	97.4	1.5	0	1.1	3843
Hb A_2_-Laibin	*HBD*:c.52A>C	1	147	82	27.3	97.5	1.4	0	1.1	4097
Hb A_2_-Hechi	*HBD*:c.347C>T	1	143	92.1	29.6	98.7	1.3	0	0	3230
Hb A_2_-Nanning	*HBD*:c.277C>T	1	144	84.8	28.9	98.7	1.3	0	0	Unregistered
Hb A_2_-Guigang	*HBD*:c.17C>T	1	81	65.9	17.5	98.7	1.3	0	0	Unregistered
Hb A_2_-Wuzhou	*HBD*:c.374C>T	1	127	88.4	28.8	98.5	1.5	0	0	Unregistered
Hb A2-Jinxiu	HBD:c.186G>T	1	142	87.1	28.5	97.6	1.4	0	1	Unregistered
Hb A2-Liuzhou	HBD:c.290T>C	1	121	85.3	28.7	98.2	1.8	0	0	Unregistered

* : One sample was amniotic fluid. N: Undetected. Reference ranges for parameters: Hb (115–155 g/L for females, 120–160 g/L for males), MCV (80–100 fL), MCH (27–34 pg), Hb A (91.5%–97.6%), Hb A2 (2.4%–3.5%), Hb F (0%–5.0%).

### Co-inherited δ-globin gene andα/β-globin gene mutations

Eleven cases harbored compound heterozygosity involving δ-globin and α/β-globin mutations, including 5 cases with α-globin mutations and 6 cases with β-globin mutations (Table 3). Co-inherited δ-globin gene and α/β-thalassemia resulted in a hematological phenotype that was comparable to the thalassemia it was associated with.

**TABLE 3:** Hematological characteristics and Hb analysis of homozygous mutation/double heterozygous mutations in δ-globin gene or coinherited δ-globin gene and α/β-globin genes.

No.	Gender	Age (years)	Hb (g/L)	MCV (fL)	MCH (pg)	Hb A (%)	Hb A2 (%)	Hb F (%)	Variant (%)	Genotypes
1	Female	45	119	67.9	20.9	97.4	1.2	1.4	0	--^SEA^/αα, δ/δ^−77^
2	Female	56	113	80	25.4	98.8	1.2	0	0	-α^4.2^/αα, δ/δ^−77^
3	Female	29	118	70.9	22	98.8	1.2	0	0	-α^4.2^/-α^4.2^, δ/δ^−77^
4	Female	24	111	69.1	21.6	98.1	1.2	0	0.7	--^SEA^/αα, δ/δ^CD43 (Hb A2-Melbourne)^
5	Male	27	158	91.9	30.3	97.1	1.1	0	1.0, 0.8	α^CS^α/αα, δ/δ^CD43 (Hb A2-Melbourne)^
6	Male	36	160	86.2	29.4	54.6	1.5	0	43.9	αα/αα, β^CD114 (Hb NewYork)^/β^IVS-II-81^, δ/δ^−77^
7	Male	36	135	58.4	18.4	94.4	8.0	0	0	αα/αα, β^CD17^/β, δ/δ^CD97^
8	Female	37	115	67.1	20.4	94	2.6	3.4	0	αα/αα, β^CD41-42^/β, δ/δ^CD97^
9	Male	28	148	60.3	18.8	95.1	2.6	0.4	1.9	αα/αα, β^CD41-42^/β, δ/δ^CD43 (Hb A2-Melbourne)^
10	Female	30	135	91.2	29.8	53.3	1.3	0	44.3, 1.1	αα/αα, β^CD114 (Hb NewYork)^/β, δ/δ^CD43 (Hb A2-Melbourne)^
11	Female	5	109	57.1	18.9	91.9	2.7	4	1.4	αα/αα, β^CD41-42^/β, δ/δ^CD46 (Hb A2−Yulin)^
12	Male	31	151	89.8	30.9	98.1	0	1.9	0	αα/αα, δ^−77^/δ^−77^
13	Female	25	118	85	26.4	97.8	0	0.6	1.6	αα/αα, δ^CD43 (Hb A2-Melbourne)^/δ^CD43 (Hb A2-Melbourne)^
14	Male	35	142	88.5	30.2	98.6	1.4	0	0	αα/αα, δ^CD87^/δ^−77^
15	Male	34	149	88.9	30.2	98.8	1.2	0	0	αα/αα, δ^CD87^/δ^−77^
16	Male	32	152	88.4	29.1	97.6	1.4	0	1	αα/αα, δ^CD116 (Hb A2-Coburg)^/δ^−130^
17	Male	35	162	91.5	31.4	98.0	1.3	0	0.7	αα/αα, δ^CD79^ ^(Hb A2-Guangxi)^/δ^polyA+70^

Reference ranges for parameters: Hb (115–155 g/L for females, 120–160 g/L for males), MCV (80–100 fL), MCH (27–34 pg), Hb A (91.5%–97.6%), Hb A2 (2.4%–3.5%), Hb F (0%–5.0%).

### Eight novel mutations of δ-globin gene

**FIGURE 2:**
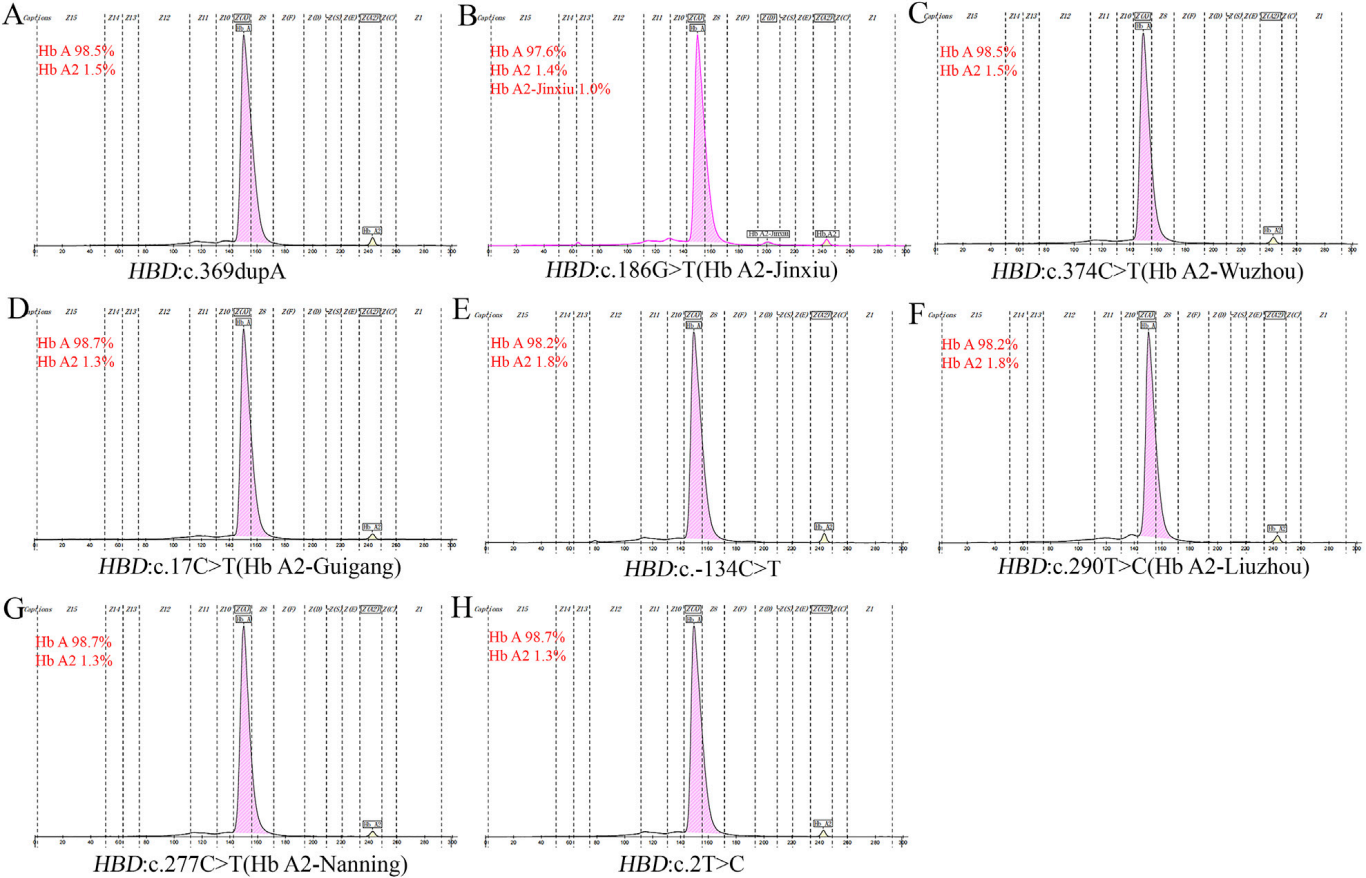
Electropherograms of eight novel mutations. **(A)** *HBD*:c.369dupA; **(B)** *HBD*:c.186G>T (Hb A_2_-Jinxiu); **(C)** *HBD*:c.374C>T (Hb A_2_-Wuzhou); **(D)** *HBD*:c.17 C>T (Hb A_2_-Guigang); **(E)** *HBD*:c.−134 C>T; **(F)** *HBD*:c.290T>C (Hb A_2_-Liuzhou); **(G)** *HBD*:c.277C>T (Hb A_2_-Nanning); **(H)** *HBD*:c.2T>C.

**FIGURE 3:**
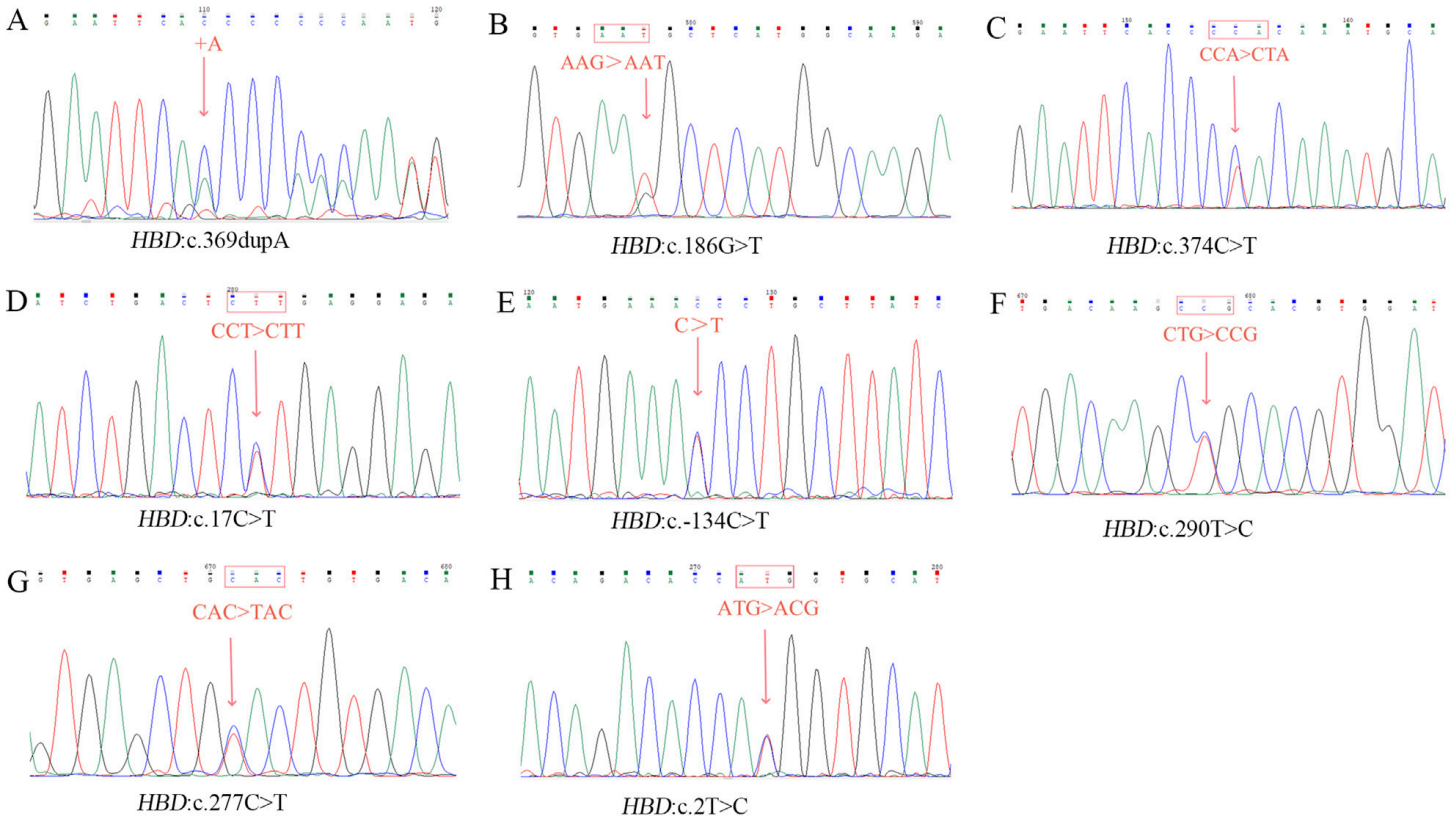
Sanger sequencing result of eight novel mutations. **(A)** *HBD*:c.369dupA; **(B)** *HBD*:c.186G>T; **(C)** *HBD*:c.374C>T; **(D)** *HBD*:c.17 C>T; **(E)** *HBD*:c.−134 C>T; **(F)** *HBD*:c.290T>C; **(G)** *HBD*:c.277C>T; **(H)** *HBD*:c.2T>C.

In this study, eitght novel δ-globin gene mutations (three δ-thalassemia and five δ-globin variants) were identified through systematic molecular and Hb analyses (Figures 2, 3). Hematological profiling presented nomal erythrocyte indices in most carriers. Quantification of Hb fractions by CE demostrated uniformly reduced Hb A_2_ levels across all variants (1.3%–1.8%). All variants were classified as novel mutations based on absence in population databases. Bioinformatic analysis showed that seven of the eight novel mutations were deleterious (Table 4).

**TABLE 4:** Bioinformatics analysis using three different predictive tools.

HGSV name	Common name	Polyphen-2	Mutation taster	SIFT
*HBD*:c.−134C>T	−84C>T	N	N	N
*HBD*:c.2T>C	Int ATG>ACG	N	Deleterious	N
*HBD*:c.17C>T	Hb A_2_-Guigang	Benign	Deleterious	Tolerated
*HBD*:c.186G>T	Hb A_2_-Jinxiu	Possibly damaging	Deleterious	Deleterious
*HBD*:c.277C>T	Hb A_2_-Nanning	Probably damaging	Deleterious	Deleterious
*HBD*:c.290T>C	Hb A_2_-Liuzhou	Probably damaging	Deleterious	Deleterious
*HBD*:c.369dupA	CD122/123(+A)	N	Deleterious	N
*HBD*:c.374C>T	Hb A_2_-Wuzhou	Possibly damaging	Deleterious	Tolerated

N : not applicable.

## Discussion

In this study, our analysis identified 28 distinct δ-globin variants and δ-thalassemia mutations among 127 carriers, corresponding to a population-level carrier frequency of 0.81% (127/15,642). The prevalence is higher than in Thailand and Tunisia, as well as that reported by other Chinese investigators (Hanart et al., 2023; Kasmi et al., 2021; Xu et al., 2023; Liu et al., 2013). The most common discovery was δ-thalassemia (81 cases, 0.52% carrier rate), which was followed by δ-globin variant (46 cases, 0.29% carrier rate). Notably, eight novel mutations were detected; comprising five δ-globin variants and three δ-thalassemia mutations, none previously cataloged in the HbVar or IthaGenes databases. The finding of eight novel mutations (6.3% of the total number of δ-globin gene mutations) further highlights the limitations of existing variant databases. This study indicates that a need for integration of δ-globin mutations screening into existing thalassemia prevetion protocols in the region.

The molecular epidemiology of δ-thalassemia in this cohort revealed a predominance of the *HBD*:c.−127T>C variant, accounting for 67.8% (61/90) of cases. This prevalence consisted with previous reports from China but exceeds Chinese regional frequencies documented in earlier studies (63.2% and 51.6%) (Xu et al., 2023; Liu et al., 2013). Surprisingly, the second most frequent mutations, Hb A_2_-Melbourne (27.8%, 25/90), demonstrated a disproportionately higher incidence compared to historical Chinese cohort data. This finding represents a notable deviation from established mutation profiles in China and may be attributed to the genetic heterogeneity of regional populations. Genotypic analysis indicated that the majority of δ-globin mutations occurred in heterozygous states, with a few co-inheritances of α/β-globin mutations. Only two isolated homozygous cases, one δ-thalassemia (*HBD*:c.−127T>C) and one δ-globin variant (Hb A_2_-Melbourne), were identified, both exhibiting undetectable Hb A_2_ peak by CE. δ-thalassemia and δ-globin variant heterozygotes, all of them showed reduced A_2_ values in their CE results (1.1%–1.8%), and some of the δ-globin variants additionally detected low levels of Hb A_2_ variant peaks. In this study, six δ-globin variants failed to produce resolvable Hb A_2_ variant peaks (Table 2). Hematological parameters of heterozygous and homozygous cases remained within normal range, reinforcing the clinically silent phenotype associated with δ-globin mutations.

Eleven cases with co-inherited δ-globin and α/β-globin mutations displayed clinically diverse symptoms ranging from asymptomatic to mild anemia. δ-globin combined with thalassemia showed a thalassemia phenotype with decreased values of hematological parameters MCV and MCH, whereas in the case of the combined α/β-globin variant, hematological parameters were normal, with separation of abnormal peaks only during electrophoresis. Elevated Hb A_2_ levels were considered as a diagnostic criterion for typical β-thalassemia trait. However, when combined with the δ-globin mutation, the Hb A_2_ level may be normal, thus masking the β-thalassemia trait and resulting in underdiagnosis. Our results emphasize the importance of integrated α/β/δ-globin genotyping in regions with high thalassemia prevalence for accurate diagnosis and counseling.

Molecular characterization confirmed all novel mutations adhere to Human Genome Variation Society (HGVS) nomenclature standards, including promoter mutations (e.g., *HBD*:c.−134C>T), initiation codon alterations (e.g., *HBD*:c .2T>C), and missense substitutions (e.g., *HBD*:c.347C>T). Bioinformatic analysis of eight novel mutations revealed diverse pathogenic potentials. *HBD*: c.277C>T and *HBD*: c.290T>C were unanimously predicted as deleterious by three tools, while the prediction of the two software programs for *HBD*: c.186G>T and *HBD*: c.374C>T were inconsistent. Surprisingly, for the prediction of *HBD*: c.17C>T, they had opposite conclusions, with Mutation Taster considered deleterious, while PolyPhen-2 and SIFT were recognized as benign and tolerated. These findings underscored the limitations of prediction tools and the need for functional studies to validate predictions, particularly for variants with atypical phenotypes. Based on the residence of the probands, we named the δ-globin variants *HBD*:c.17C>T, *HBD*:c.186G>T, *HBD*:c.277C>T, *HBD*:c.290T>C, and *HBD*:c.374C>T as Hb A_2_-Guigang, Hb A_2_-Jinxiu, Hb A_2_-Nanning, Hb A_2_ -Liuzhou, Hb A2-Wuzhou, respectively. In addition, *HBD*: c.347C>T has been registered in the Ithagene database, but did not name the Hb name; we try to name it as Hb A_2_-Hechi to facilitate the study and communication.

**FIGURE 4:**
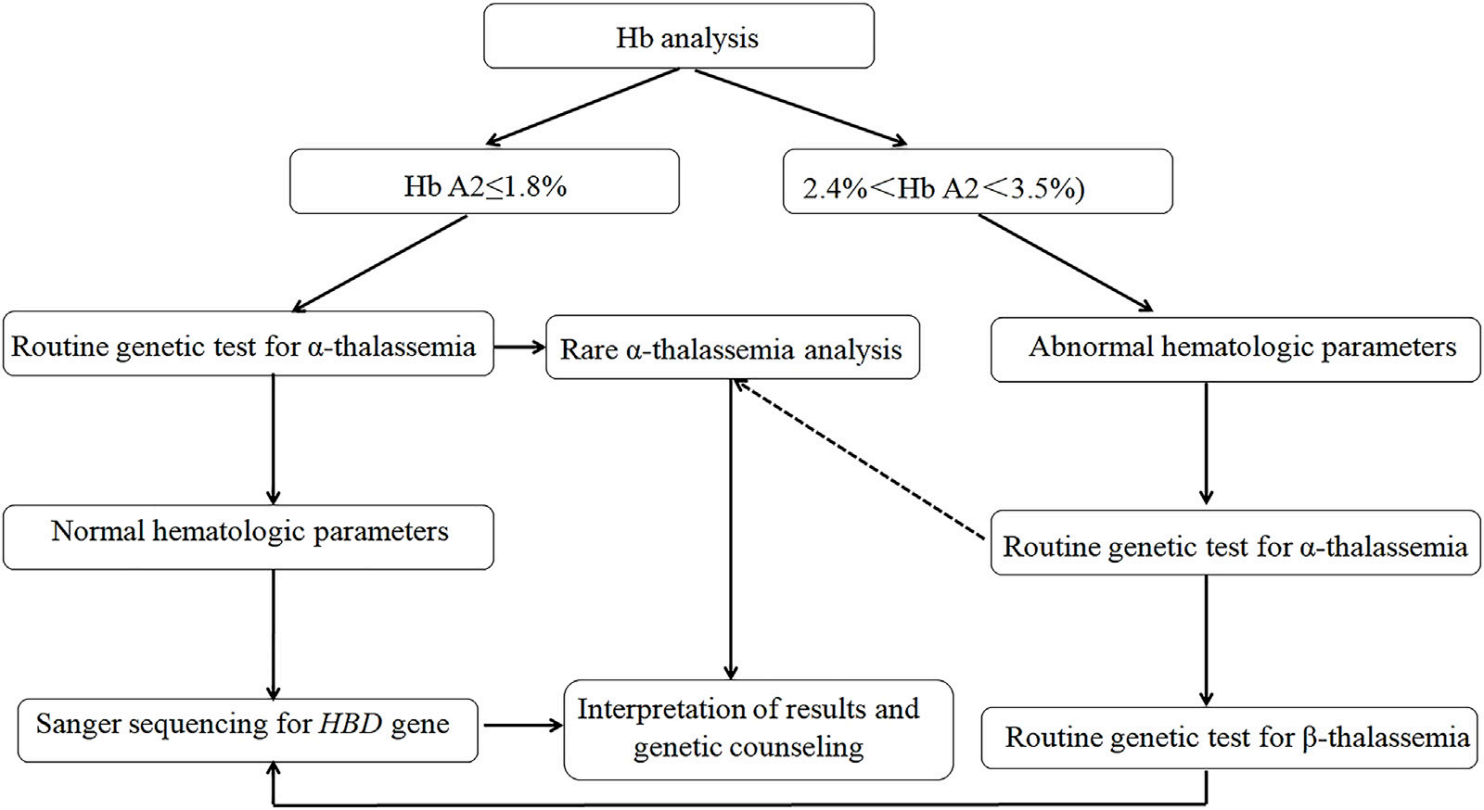
Recommended workflow for the δ-globin gene mutations.

This study expanded the molecular spectrum of δ-globin gene defects and highlighted critical discrepancies in mutation frequencies across populations, underscoring the necessity for ethnically tailored genetic databases to optimize hemoglobinopathy diagnostics. While our findings did not represent a comprehensive epidemiological survey of δ-globin molecular defects in China, they revealed a substantial carrier burden (0.81% aggregate frequency) within the studied cohort, challenging historical perceptions of δ-globin variants as clinically negligible in Chinese populations. To address diagnostic challenges, we implemented a standardized δ-globin screening protocol (Figure 4) integrating CE, multiplex gap-PCR, andSanger sequencing, which successfully resolved β-thalassemia cases masked by conventional screening methods, thereby mitigating risks of underdiagnosis. The workflow’s efficacy in detecting co-inherited α/β-globin defects (8.7% of resolved cases) demonstrated its utility for refining carrier risk stratification and informing precision prenatal counseling.

## Data Availability

The datasets presented in this study can be found in online repositories. The names of the repository/repositories and accession number(s) can be found in the article/supplementary material.
